# Unpacking Young Adults’ Fact-Checking Intent on Oral Health Misinformation: Parallel Mediating Roles of Need for Cognition and Perceived Seriousness—A Cross-Sectional Study

**DOI:** 10.3390/healthcare13111354

**Published:** 2025-06-05

**Authors:** Donghwa Chung, Yongjun Zhang, Jiaqi Wang, Yanfang Meng

**Affiliations:** 1 School of Journalism and Communication, Central China Normal University, Wuhan 430079, China; chungdonghwa@ccnu.edu.cn (D.C.); jiaqiwang@mails.ccnu.edu.cn (J.W.); 2 School of Journalism and Communication, Beijing Institute of Graphic Communication, Beijing 102699, China; mengyf@bigc.edu.cn

**Keywords:** oral health misinformation, fact-checking intent, status quo bias theory, young adults, survey

## Abstract

Background: Oral health misinformation has increasingly proliferated across social media platforms in China, prompting rising concerns about the accuracy of health-related content. Fact-checking intent has been identified as a key strategy for mitigating the spread of such misinformation. However, empirical research on the psychological factors shaping engagement in fact-checking behaviors remains limited. Objectives: This study aims to examine the association between misinformation recognition and fact-checking intent among Chinese young adults (aged 18–36). Methods: Guided by status quo bias theory, this study integrates psychological constructs into its theoretical framework. A stimulus-based online survey was conducted, yielding 452 valid responses. Direct, mediated, and serial mediation hypotheses were tested using SPSS 26.0 and Jamovi 2.6.24. Results: The findings indicate a significant positive relationship between misinformation recognition and fact-checking intent. A parallel mediation model involving need for cognition and perceived seriousness was supported, clarifying the psychological mechanisms underlying this relationship. Conclusions: This study contributes valuable empirical evidence to the understanding of fact-checking intent among Chinese young adults in the context of oral health misinformation. The findings offer practical implications for developing targeted interventions to increase misinformation awareness and promote active engagement in fact-checking behaviors.

## 1. Introduction

In recent years, with the substantial advancement of media technology, the acquisition of health-related knowledge through social media platforms has become increasingly significant [1]. Social media has emerged as a major source for individuals to access and engage with health information, leading to greater willingness to share [2], comment on [3], and discuss topics [4] such as vaccination, diet, and oral health management [5,6,7]. While experts and scholars acknowledge the potential of social media platforms to promote health recognition and encourage positive health behaviors, persistent concerns remain regarding the reliability of the information disseminated, particularly in relation to health misinformation [8]. Health misinformation is commonly defined as inaccurate or misleading health-related information that lacks scientific validation or is based on unverified evidence [9]. Due to the widespread reach and unregulated nature of social media, misinformation can rapidly propagate, making it difficult for individuals to distinguish credible information from false claims [10]. This, in turn, increases the risk of improper self-treatment and mismanagement of health conditions [11].

Existing evidence indicates that health-related misinformation is proliferating across major social media platforms worldwide. This issue is equally severe in China, where addressing health misinformation has become a public health priority [12]. Of particular concern is the increasing prevalence of oral health misinformation, which poses a significant challenge to public health. According to the Beijing Advertising Monitoring Report, 25% of misleading health advertisements were disseminated by 1600 media platforms [13]. The rapid escalation of oral health misinformation since late 2023 underscores the urgent need for targeted interventions [14]. Such misinformation encompasses a wide range of false or misleading claims, including unverified do-it-yourself dental remedies and advertisements promoting low-cost dental implants from unlicensed clinics [15,16].

Previous reports suggest that Chinese young adults exhibit a higher tendency to purchase health and beauty products online compared with other demographic groups [17]. However, this trend has also heightened their vulnerability to oral health-related fraud. Young adults, in particular, have been disproportionately targeted by such deceptive practices [16,18]. For example, the use of unregulated tooth-whitening products often purchased online has been associated with adverse outcomes, including damage to the gums and lips [18]. The growing spread of oral health misinformation has drawn increasing attention to the need for effective risk mitigation strategies.

One promising intervention for addressing misleading health information is the implementation of fact-checking mechanisms [19]. These include verifying inaccurate claims, issuing corrections, and conducting manual fact-checking strategies that play a critical role in mitigating the spread of misinformation. Among these, manual fact-checking stands out as particularly valuable as it facilitates deeper interpretation and critical evaluation of content [20]. In response to the growing need, this study explores strategies to strengthen individuals’ ability to independently evaluate the credibility of health information. In this context, fact-checking intent (FCI) is defined as an individual’s willingness to critically assess and verify health-related claims to counter oral health misinformation. This process may involve evaluating the credibility of information sources, consulting peers, and analyzing content circulated on social media platforms.

In light of these pressing issues, this study examines oral health misinformation based on a threefold rationale. First, the rising prevalence of false oral health information in China underscores the urgent need for research to mitigate its risks, prompting increased scholarly attention. Second, although research has examined the types, spread, and countermeasures of online health misinformation [21,22], the nuanced effects on fact-checking intent in the context of oral health, particularly among young adults, remain largely underexplored. Third, although growing research presents mixed findings on the association between false health information exposure and fact-checking intent in Western contexts [23,24], empirical evidence remains limited for oral health misinformation in collectivist societies like China.

Amid growing concerns over the spread of oral health misinformation in China, this study systematically investigates the association between misinformation recognition (MR) and FCI among Chinese young adults (aged 18–36). The study is grounded in status quo bias theory, which has been extensively applied and empirically tested in the context of health behavior change [25,26]. The theoretical framework further incorporates the need for cognition and perceived seriousness to examine the direct, mediated, and serially mediated pathways underlying this relationship. The findings contribute to a more nuanced understanding of the complex association between MR within the oral health domain and FCI in this population. Additionally, the study offers practical insights for enhancing awareness of oral health misinformation and promoting greater engagement in fact-checking behaviors.

## 2. Literature Review

### 2.1. Theory of Status Quo Bias

Status quo bias (SQB) theory posits that individuals tend to prefer maintaining their current situation rather than making changes [27]. This theory provides a critical framework for understanding decision-making processes, particularly the factors that inhibit change [28]. Scholars have extensively explored SQB, highlighting that a common mechanism through which an option becomes the status quo is its designation as the default [29]. When presented with a default option, individuals often exhibit reluctance to switch due to the perception of potential loss, a tendency grounded in loss aversion [30]. This phenomenon is closely linked to the endowment effect, wherein individuals assign greater value to something they already possess than to alternatives they do not own [31]. Furthermore, individuals frequently adhere to the status quo due to perceived switching costs, even when such costs are objectively minimal. As a result, they often continue with a default option despite the availability of superior alternatives [32].

In recent decades, SQB theory has been widely applied across various domains, including technology adoption [33], online purchasing behavior [28], and public health [34]. Within the health behavior literature, SQB has been predominantly associated with unhealthy behaviors, such as poor dietary habits and ineffective weight management [35]. However, emerging evidence suggests that reversed status quo bias (RSQB) may also play a role in facilitating health-promoting behaviors. RSQB refers to individuals’ tendency to actively reject habitual behaviors and adopt preventive health measures, particularly in response to external stimuli such as health messages. For instance, during the COVID-19 pandemic, individuals demonstrated a proactive approach to prevention, adhering strictly to health guidelines and engaging in protective behaviors following exposure to public health messages [36].

Additionally, prior research has identified a two-pronged approach to health behavior change, wherein individuals not only break existing unhealthy habits but also develop new, sustainable health behaviors, such as maintaining weight control [33]. This aligns with the present study’s focus, which posits that RSQB is a key factor in motivating individuals to reject default behaviors and actively engage in fact-checking, particularly in response to misleading information within the oral health context. However, research on the associated relationship between RSQB and FCI within collectivistic cultural contexts (e.g., China) remains limited. Given this critical gap, the present study further investigates the role of RSQB in the relationship between MR and FCI among Chinese young adults.

### 2.2. Oral Health Misinformation in Short Videos and Its Impact in China

Advancements in digital technology have expanded new media, making social media a key platform for health information dissemination through text, videos, and blogs [37]. However, alongside its benefits, social media also facilitates the spread of misleading health information. In China, the Three-Year Action Plan (2024–2027) highlights the importance of leveraging digital media for public health communication. The initiative encourages government agencies and health experts to disseminate high-quality, authoritative health content through media platforms [38]. Among these, short-form video (such as Douyin) has gained prominence, engaging both the public and healthcare professionals by rapidly capturing attention and raising disease awareness [39]. Despite its advantages, health misinformation is a growing concern on short-form video platforms, particularly in China. Weak identity verification and lack of gatekeeping allow individuals to falsely present themselves as medical professionals, as seen on Douyin (China’s TikTok). This has led to the widespread dissemination of inaccurate cancer-related information, exacerbating misinformation risks and underscoring the need for stricter content regulation [39].

The growing prevalence of oral health misinformation on short-form video platforms presents a significant public health concern. Unverified claims, such as false information about dental implants and the substitution of mouthwash for brushing, spread rapidly and contribute to harmful oral health practices [40]. Given the potential risks associated with misleading oral health content in short-form videos, Chinese young adults are particularly vulnerable to digital misinformation, increasing their risk of adopting ineffective or detrimental health behaviors.

Given the urgency of this issue, the present study investigates MR and FCI among Chinese young adults following exposure to misleading oral health content in short-form videos. Employing a stimulus-based approach, this research explores the dynamics of trending oral health misinformation and the corresponding engagement and responses among this demographic. In doing so, the study offers insights into the nuanced relationships between misinformation and fact-checking intent within the context of digital health communication.

### 2.3. Exploring the Relationships Among MR, NFC, and PS

In the context of health communication on social media, health issue recognition has been shown to predict individuals’ engagement in health-related behaviors [41]. Recognition is generally defined as an individual’s familiarity with information, particularly their capacity to recall previously encountered events or details [42]. More recently, health misinformation recognition has been conceptualized as the cognitive process of identifying and discerning misleading or false health information. The present study further refines this concept by focusing specifically on the type of misinformation encountered. Accordingly, MR refers to individuals’ ability to evaluate misleading short-form videos related to oral health and to determine whether the information presented is false or unreliable.

An increasing number of studies have examined how individuals process information, either analytically or heuristically. Need for cognition (NFC) is a key aspect of analytical processing, reflecting a dispositional tendency to engage in deep reflection. Previous research suggests that individuals think more critically when exposed to health-related information. Borah (2022) conceptualized NFC as a preference for systematic information processing that enhances analytical engagement with health messages [43]. In line with this established definition, the present study examines NFC in the context of processing misleading oral health information.

A widely recognized definition of perceived seriousness (PS), also referred to as perceived severity, is the extent to which an individual associates a health event or its consequences with negative health outcomes (e.g., a cancer diagnosis) [44]. Such perceptions can heighten awareness of potential or existing health conditions, influencing individuals’ risk perception and health-related decision-making [45,46]. Drawing on a review of relevant health studies, the present study defines PS as the extent to which Chinese young adults perceive oral health issues as severe. This perception, in turn, correlates with their engagement in specific preventive behaviors (e.g., FCI).

Prior research has identified awareness of green messaging as a critical antecedent influencing individuals’ emotional and functional value, both of which are associated with NFC [47]. Furthermore, studies suggest that greater awareness of sustainability issues is positively correlated with increased effortful thinking [48], commonly referred to as NFC. Additionally, previous research has demonstrated that recognition of mental health issues is positively correlated with individuals’ ability to accurately identify and report mental health symptoms [49]. This indicates that higher recognition of an issue enhances individuals’ analytical engagement with the topic, leading to more systematic processing and critical evaluation. Furthermore, a considerable body of research has examined the association between individuals’ recognition of PS and health threats. For instance, Mya et al. (2020) found that awareness of respiratory infectious diseases and their associated risks influenced individuals’ perceived severity of the disease [50]. In addition, Limbu et al. (2022) confirmed that higher health recognition, such as knowledge of the disease and prior diagnosis, was correlated with lower vaccine hesitancy, suggesting that individuals with greater health awareness tend to take the disease more seriously [51]. This is because when awareness of vaccine intake arises, their PS of the situation also increases. Based on these findings, the following hypotheses are proposed:

**Hypothesis** **1.** *MR is positively associated with Chinese college young adults’ NFC.*

**Hypothesis** **2.** *MR is positively associated with Chinese college young adults’ PS.*

### 2.4. Parallel Mediation Model: The Roles of NFC and PS

As discussed in the previous section, empirical evidence suggests that NFC and PS are predicted by MR. However, an increasing body of research has also examined NFC and PS as antecedents that contribute to individuals’ preventive behaviors. Taken together, these insights point to the potential for the two psychological factors to serve as parallel mediators in the relationship between MR and FCI among Chinese young adults. For instance, a prior empirical study found that students with greater recognition of sustainability issues tend to exhibit higher levels of critical thinking regarding sustainability [48], indicating a potential association between MR and NFC. Furthermore, engagement in analytical processing of online health information (e.g., NFC) has been shown to be positively associated with individuals’ participation in health prevention behaviors [52]. Additionally, recognition of mental health issues has been identified as a key predictor of individuals’ ability to accurately identify and report mental health symptoms [49]. Moreover, a greater preference for systematic analytical engagement with health issues has been linked to increased adoption of healthy behaviors [53].

Previous public health research has confirmed that greater awareness of highly contagious respiratory infections is associated with higher perceived severity of the disease [50]. Furthermore, perceived severity (also referred to as PS) has been found to be positively associated with engagement in health behaviors during disease outbreaks [54]. Similarly, an empirical study reported that recognition of a health issue is significantly associated with higher perceived severity [51]. Additionally, perceived severity has been found to be positively associated with individuals’ participation in preventive behaviors [55]. Building on these findings, it is plausible that higher MR increases NFC and PS which, in turn, may strengthen FCI among Chinese young adults. Thus, the following hypotheses are proposed:

**Hypothesis** **3.** *NFC mediates the relationship between MR and Chinese young adults’ FCI.*

**Hypothesis** **4.** *PS mediates the relationship between MR and Chinese young adults’ FCI.*

### 2.5. The Serial Mediating Role of Psychological Factors and RSQB

A growing body of literature has produced mixed findings regarding individuals’ tendency to actively change habitual unhealthy behaviors and adopt preventive measures [35,56]. These findings have led to broader investigations of RSQB in public health contexts. While prior studies proposed a parallel mediation model involving NFC and PS in the relationship between MR and Chinese young adults’ FCI, emerging evidence suggests a potentially more complex serial mediation process. For example, one study found that recognition of sustainability issues was significantly and positively associated with NFC [48]. Moreover, deep engagement with unfamiliar messages, which involves careful and effortful evaluation of information and is aligned with NFC, is more likely to disrupt established habits, also referred to as RSQB [57]. This, in turn, increases individuals’ willingness to engage in preventive health behaviors [35].

Recent empirical evidence has also suggested that PS and RSQB may function as a serial mediation mechanism in the relationship between MR and FCI. For example, recognition of health issues has been found to be positively associated with individuals’ PS [50]. Additionally, PS has been positively associated with RSQB [58]. Furthermore, breaking unhealthy weight-related habits, equivalent to RSQB, has been shown to increase individuals’ likelihood of engaging in preventive health behaviors, such as effective weight management [56]. Taken together, these findings provide compelling empirical evidence supporting the existence of serial mediation mechanisms in this nuanced relationship. Therefore, this study proposes the following serial multiple mediation model: NFC → RSQB and PS → RSQB. Accordingly, the following hypotheses are proposed:

**Hypothesis** **5.** *NFC and RSQB play a serial mediating role in the relationship between MR and Chinese young adults’ FCI.*

**Hypothesis** **6.** *PS and RSQB play a serial mediating role in the relationship between MR and Chinese young adults’ FCI.*

**Figure 1:**
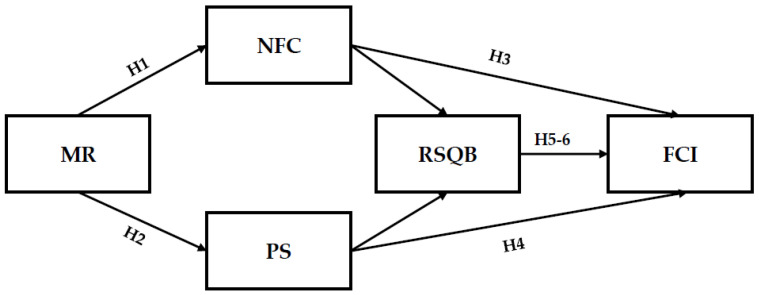
A hypothesized model of factors associated with young adults’ fact-checking intent.

This study proposes a hypothesized model to illustrate the relationships among the key variables (see Figure 1).

## 3. Methods

### 3.1. Measurements

The survey measurements employed in this study were adapted and refined based on established instruments from prior research [43,59,60,61,62,63,64] and encompassed six scales: MR, NFC, PS, RSQB, and FCI. Recognizing that measurement variability may occur across different cultural and national contexts, modifications were conducted through a two-step process, consistent with operationalization practices commonly reported in the literature. First, to enhance construct validity, three language experts independently translated the original English instruments into Chinese [65]. Second, two public health experts evaluated the translated items for content and face validity to ensure both linguistic accuracy and conceptual consistency with the original constructs. The final measurement model, including factor loadings, composite reliability (CR), and average variance extracted (AVE) for each item, is presented in Appendix A.

A pilot study was conducted as a critical step to evaluate the reliability and validity of the adapted measures across different contexts [66]. Following established procedures [67], on 21 January 2025, sixty-five volunteers (aged 18 to 36) were recruited via various social media platforms, including Xiaohongshu, to participate in an independently conducted pilot study. Data obtained during this phase were excluded from the main analysis to avoid analytical bias and to uphold the quality and integrity of the final dataset. At the beginning of the online questionnaire, participants were instructed to watch two short videos (each under 40 s) related to oral health misinformation. The video selection process was guided by methodologies used in prior research [68,69]. First, the top 100 videos associated with oral health misinformation were identified on Douyin, one of the most widely used short-form video platforms in China. Second, five representative videos were shortlisted based on the number of likes and shares. Finally, four health communication specialists independently evaluated the shortlisted videos and reached a consensus on the two most suitable clips for the study. The main characteristics of the selected videos are presented in Table 1.

Following the pilot study procedure, participants were asked to identify any ambiguous survey items and provide qualitative feedback. The pilot results demonstrated acceptable internal consistency, with Cronbach’s alpha values ranging from 0.84 to 0.92. In addition, the Kaiser–Meyer–Olkin (KMO) measure and Bartlett’s test of sphericity provided evidence supporting construct validity. Structural integrity of the revised scales was further confirmed, as all factor loadings exceeded the recommended threshold of 0.50. Following the guidelines provided by Eysenbach’s Checklist for Reporting Results of Internet E-Surveys (CHERRIES) [70], the current study includes the completed checklist in the Supplementary File S1, detailing the steps carried out during the research process.

### 3.2. Data Collection Procedures

The formal participant recruitment was conducted through Tencent Questionnaires (https://wj.qq.com/, accessed on 25 January 2025), a widely utilized survey platform for empirical studies in China [71]. The platform hosts a verified pool of over 48 million Chinese individuals with authenticated identities. Ethical approval for the study was granted by the Academic Committee of the School of Journalism and Communication at Central China Normal University (CC20250120). Written informed consent was obtained at the beginning of the online questionnaire, and assurances of confidentiality were provided to all participants. Individuals were informed of the study’s objectives and retained the right to withdraw at any stage. Eligibility criteria required participants to (1) be between 18 and 36 years of age and (2) actively use major social media platforms such as WeChat, Douyin, and Xiaohongshu. Individuals who did not use these platforms were excluded from participation. Recruitment began on 25 January 2025, and ended on 10 March 2025. A total of 510 Chinese young adults were invited to participate, with 482 individuals completing the survey.

To ensure data quality, two data-cleaning procedures were applied: (1) exclusion of responses submitted in under 120 s (N = 18) and (2) exclusion of straight-line response patterns (N = 12). The final analytical sample comprised 452 valid responses. The required sample size was determined using G*Power 3.1, a widely utilized tool in scholarly research across various disciplines [72]. For the present study, a significance level of 0.05, a statistical power of 0.85, and an effect size of 0.15 were specified with four predictor variables. The results indicated that a minimum sample size of 95 was necessary. To further evaluate the sample size requirements for the mediated hypothesis model, the present study employed Monte Carlo power analysis, a method widely recognized in empirical research [73]. This analysis specified a target power of 0.80, with the sample size ranging from 50 to 300, using 1000 replications and a 95% confidence interval. The findings suggested that the required sample size ranged between 210 and 295. Given that the current study employs a sample size of 452, it exceeds these recommendations, thereby ensuring sufficient statistical power for hypothesis testing.

Evaluating discriminant validity is a critical step in ensuring that measurement constructs accurately capture the intended concepts [74]. Therefore, this study employs the Fornell–Larcker criterion, which has been widely applied in previous research [75]. As shown in Table 2, the square root of each AVE score surpassed the corresponding correlations with other variables, indicating that the assessment of discriminant validity meets an acceptable standard.

While the Fornell–Larcker criterion is widely used to assess discriminant validity, it has been criticized for relying on AVE and loadings, which may overlook validity issues. The HTMT ratio offers a more robust, correlation-based alternative [74]. This study applied HTMT to five variables, with the results being presented in Table 3. The HTMT criterion suggests that values should be below 0.85 or 0.90 to confirm that variables are distinct within the dataset [76]. In this study, all HTMT values fall below these thresholds, indicating no issues with discriminant validity.

Common method bias (CMB) is a critical concern in cross-sectional research, requiring rigorous assessment before hypothesis testing [77]. This evaluation examines whether systematic error arises due to the use of similar survey methods across variables. To address this issue, Harman’s one-factor test was conducted. The results showed that a single factor accounted for 32.22% of the variance, below the 50% threshold. Thus, common method bias is not a concern in this study.

Confirmatory factor analysis (CFA) is essential for examining whether a set of observed variables accurately represents the underlying theoretical or conceptual factors. Following the methodology of prior studies, a one-factor model was specified and evaluated for model fit across key variables [78]. The analysis was conducted using Jamovi 2.6.24. The results indicated an acceptable model fit, with χ^2^/df = 3.29, CFI = 0.92, TLI = 0.91, and RMSEA = 0.07, with a 90% confidence interval ranging from 0.06 to 0.07. These findings suggest that the model provides a satisfactory representation of the data.

### 3.3. Data Analysis Methods

Data analysis was conducted using SPSS 26.0 and Jamovi 2.6.24. In SPSS, the following analyses were performed: Cronbach’s alpha reliability test, common method bias (CMB) assessment, Fornell–Larcker criterion, HTMT ratio, descriptive statistics, and serial mediation analysis. Jamovi was used for confirmatory factor analysis (CFA) and structural equation modeling (SEM). Model fit was evaluated using multiple goodness-of-fit indices, including χ^2^/df, GFI, RNI, CFI, and RMSEA.

## 4. Results

### 4.1. Descriptive Data

A total of 452 valid participants’ responses were collected. The key demographic characteristics of the respondents are presented in Table 4.

The majority of the sample were female (N = 243, 53.8%). Most participants were either undergraduate students (N = 318, 70.4%) or postgraduate students (N = 111, 24.6%). Additionally, the largest age group ranged from 18 to 23 years (N = 292, 64.6%), followed by those aged 24 to 28 years (N = 106, 23.5%). In terms of monthly income, the most common ranges were CNY 9000–13,999 (N = 131, 29.0%) and CNY 1000–3999 (N = 119, 26.3%).

### 4.2. Path Analysis Tests

**Figure 2:**
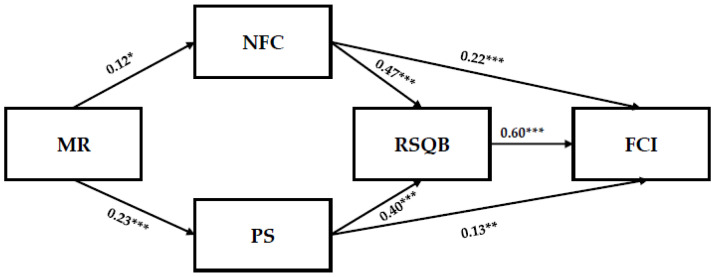
A hypothesized model of factors associated with young adults’ fact-checking intent. * *p* < 0.05, ** *p* < 0.01, *** *p* < 0.001.

Path analysis was conducted using Jamovi 2.6.24 to test Hypotheses 1 and 2. The analysis specified NFC, PS, RSQB, and FCI as endogenous variables, while MR and control variables (sex, age, and education) were included as exogenous variables. Model fit indices indicated an excellent fit to the data (χ^2^/df = 1.35, GFI = 0.99, RNI = 0.99, CFI = 0.99, RMSEA = 0.03). The path coefficients are presented in Figure 2.

For the first direct pathway (Hypothesis 1), MR explained 41% of the variance in NFC (R^2^ = 0.41, *p* < 0.05) and exhibited a significant positive relationship (β = 0.12, t = 2.50, *p* < 0.05, 95% CI [0.12, 0.26]). In the second pathway (Hypothesis 2), MR accounted for 58% of the variance in PS (R^2^ = 0.58, *p* < 0.001), also demonstrating a significant positive association (β = 0.23, t = 5.03, *p* < 0.001, 95% CI [0.13, 0.30]). These findings provide empirical support for Hypotheses 1 and 2.

To test Hypotheses 3 and 4, mediation analysis was conducted using Hayes’ PROCESS macro (Model 4) in SPSS. Bootstrapping with 2000 resamples was applied to obtain bias-corrected 95% confidence intervals for estimating indirect effects. The results for the first mediation pathway indicated that NFC mediated the relationship between MR and FCI (β = 0.10, *p* < 0.001, 95% CI [0.01, 0.10]). Similarly, the second mediation pathway demonstrated that PS served as a mediator between MR and FCI (β = 0.10, *p* < 0.001, 95% CI [0.04, 0.11]). These findings provide empirical support for Hypotheses 3 and 4.

Lastly, Hayes’ PROCESS macro (Model 6) in SPSS was used to test the serial mediation hypotheses (Hypotheses 5 and 6). The results for the first serial mediation pathway indicate that MR was positively associated with NFC (β = 0.12, t = 2.52, *p* < 0.05). NFC, in turn, was correlated with RSQB (β = 0.47, t = 13.02, *p* < 0.001) and FCI (β = 0.22, t = 6.37, *p* < 0.001). Additionally, RSQB was found to be positively associated with FCI (β = 0.51, t = 13.10, *p* < 0.001), and the overall serial mediation effect was significant (β = 0.03, t = 5.60, *p* < 0.001, 95% CI [0.01, 0.10]). For the second serial mediation pathway, MR was positively associated with PS (β = 0.22, t = 5.03, *p* < 0.001) which, in turn, was positively associated with RSQB (β = 0.40, t = 8.25, *p* < 0.001) and FCI (β = 0.13, t = 3.60, *p* < 0.01). RSQB was also positively associated with FCI (β = 0.60, t = 16.12, *p* < 0.001), and the serial mediation association was statistically significant (β = 0.10, t = 4.90, *p* < 0.001, 95% CI [0.02, 0.10]). In conclusion, Hypotheses 5 and 6 were supported. A summary of the mediated effects among variables is presented in Table 5, while Table 6 provides an overview of the hypothesis testing results.

## 5. Discussion

This study is grounded in status quo bias theory and integrates psychological factors into its theoretical framework to systematically examine how misinformation recognition in the oral health domain influences fact-checking intent among Chinese young adults (aged 18–36) in an environment where misleading health information is widespread on social media. Given the rapid and extensive dissemination of oral health misinformation in China, this research aims to rigorously explore the underlying mechanisms of this relationship, including both direct and serial mediation effects of psychological factors. This study uniquely adopts a stimulus-based approach to examine the real-world impact of socially salient oral health misinformation on this demographic, assessing their engagement with and responses to such content. The findings offer valuable insights for enhancing fact-checking behaviors and designing targeted health communication strategies for Chinese young adults.

Considering the direct effect findings, this study specifically examined the dual direct effects of misinformation recognition on need for cognition and perceived seriousness, an empirical relationship that has not yet been fully explored in oral health-related research. First, previous empirical studies have suggested that greater recognition of health-related issues enhances individuals’ engagement in effortful thinking, commonly referred to as need for cognition [49]. Consistent with prior research, the present study provides evidence that misinformation recognition reinforces the need for cognition among Chinese young adults, suggesting a preference for systematic information processing that promotes analytical engagement with health messages. Second, prior research has established a positive association between awareness of diseases and perceived seriousness, such as in the case of respiratory infectious diseases [50,51]. While recognition of diseases like COVID-19 often heightens risk perception, the impact of widespread oral health misinformation on perceived seriousness remains unclear. This study found that higher misinformation recognition is positively associated with perceived seriousness among Chinese young adults, highlighting the need for further research into the psychological mechanisms underlying this relationship. One of the key findings of this study is that, among the dual direct effects, misinformation recognition shows a stronger association with perceived seriousness than with need for cognition, as indicated by a higher R^2^ value (R^2^ = 0.58), a larger standardized coefficient (β = 0.23), and a higher t-value (t = 5.03). This suggests that Chinese young adults exhibit heightened cognitive activation and attentiveness when exposed to biased oral health information, particularly in assessing its severity. A potential explanation is that health misinformation often triggers emotional responses, which can hinder immediate cognitive deliberation. Therefore, interventions such as oral health fact-checking initiatives should integrate strategies that balance emotional engagement with analytical thinking to reduce both misinformation acceptance and unnecessary alarmism.

The parallel mediation model involving need for cognition and perceived seriousness helps explain the psychological mechanisms through which misinformation recognition is associated with fact-checking intent among Chinese young adults. First, the results indicate that need for cognition serves as a mediator in this relationship. When Chinese young adults recognize oral health misinformation, their cognitive attentiveness is activated. This engagement increases their motivation to verify information, such as by searching social media, consulting peers, or referring to credible news sources. Building on these findings, the current study extends the application of need for cognition to the oral health domain, particularly within collectivist cultural contexts such as China, which remain underexplored in existing research. Perceived seriousness was also identified as a mediating variable between misinformation recognition and fact-checking intent. This finding aligns with earlier research suggesting that recognizing health threats, such as contagious diseases, increases individuals’ perceptions of severity. Prior studies have shown that higher perceived seriousness is associated with a greater willingness to engage in protective health behaviors, especially during public health crises [50,54].

Beyond the parallel mediation model, the study examined a serial mediation pathway to better understand how misinformation recognition is correlated with fact-checking intent. Specifically, it tested whether need for cognition and perceived seriousness lead to fact-checking intent via reversed status quo bias as a second-stage mediator. The results showed that both need for cognition and perceived seriousness were significantly associated with reversed status quo bias, which in turn was positively associated with fact-checking intent. This suggests that reversed status quo bias may serve as a cognitive mechanism through which misinformation recognition prompts individuals to shift from passive information acceptance to more deliberate verification efforts.

However, it is important to note that the size of the indirect effects, particularly in the first serial mediation pathway, was relatively small. One indirect effect had a coefficient of 0.03, indicating a modest effect despite statistical significance. This suggests that future studies should consider larger sample sizes to improve the precision and reliability of mediation estimates by increasing statistical power [79]. Despite the modest effect sizes, the findings support the need to expand the theoretical frameworks used to understand fact-checking intent, particularly in the context of oral health misinformation. Models such as the health belief model and information overload theory may offer further insight. Future research should continue to explore these serial mediation pathways, especially in high-stress environments, to better understand how cognitive and cultural factors shape individuals’ responses to oral health-related misinformation in China.

Building on prior empirical work, several studies have applied theoretical frameworks to explain fact-checking intent in public health contexts in China, including protection motivation theory [80], the O-S-O-R model [81], and the influence of presumed media influence model [82]. These theoretical frameworks elucidate cognitive, emotional, and normative mechanisms, respectively, and have demonstrated effectiveness in predicting fact-checking intent among Chinese populations. While these perspectives offer important insights, they may not fully capture how individuals overcome habitual tendencies to adopt preventive behaviors, particularly when motivated to resist the status quo. To address this gap, the present study applies reversed status quo bias as a mediating mechanism linking need for cognition and perceived seriousness to Chinese young adults’ fact-checking intent. This finding offers a novel theoretical lens, particularly relevant in the context of widespread oral health misinformation. Furthermore, grounded in a psychological framework, future research could benefit from integrating information processing theories to better explain fact-checking intent regarding oral health misinformation. Cognitive load theory, for instance, highlights how elevated cognitive load can hinder preventive behaviors such as health information seeking [83]. When integrated with status quo bias, this perspective may offer a more nuanced understanding of the cognitive mechanisms underlying oral health prevention.

In addition, the current study makes several important theoretical contributions. First, while status quo bias theory has been widely applied across various contexts [84,85,86], its reversed form in relation to fact-checking intent has been largely overlooked in previous research. This study addresses that gap by providing evidence of the role that the reversed effect of status quo bias plays in shaping fact-checking intent, particularly within the context of oral health domains. Second, this study is among the first to examine fact-checking intent among young Chinese adults, a demographic recognized as vulnerable to health misinformation. By focusing on this group, this study offers new insight into how cognitive and behavioral responses to misinformation may vary across cultural and demographic contexts.

The findings of this study have important implications for addressing oral and general health misinformation. These insights are not limited to the Chinese context but are also relevant to global public health communication. Fact-checking intent showed acceptable reliability and validity, yet participants reported only modest intent to verify health information. This highlights the need for targeted campaigns that encourage critical evaluation, using engaging content across both Chinese platforms and global channels such as YouTube, Facebook, and TikTok. Second, the results showed that misinformation recognition had a stronger association with perceived seriousness than with need for cognition. This suggests that interventions should aim to balance emotional engagement with analytical thinking to both reduce susceptibility to misinformation and avoid unintended alarmism. Third, promoting the use of advanced fact-checking tools by individuals is essential. For example, a recent study highlights the potential of generative language models, such as ChatGPT-4o, in assisting with bias mitigation and offering complementary perspectives during the fact-checking process [87]. These models have shown promise in supporting efforts to counter misinformation. Given that many Chinese individuals report positive attitudes toward and perceived benefits of health-related artificial intelligence technologies [88], integrating these tools into strategies may help individuals verify oral health misinformation more effectively and respond to it in a timely manner.

This research has several limitations, which fall into three categories. First, as the study employed a cross-sectional design, it was unable to establish a causal relationship between misinformation recognition and fact-checking intent among Chinese young adults. Future research is encouraged to adopt experimental or longitudinal designs to more effectively examine causal effects. Second, although the stimulus materials were carefully selected by experts to ensure that the misleading information was both attention-grabbing and of high quality, the study measured only participants’ immediate reactions. The psychological and behavioral changes that may develop over time remain underexplored. To address this gap, future studies should consider employing longitudinal methods, such as video diary techniques, to gain deeper insight into the long-term impact of misinformation exposure. Lastly, the relatively small sample size presents a limitation. In structural equation modeling, small samples can compromise the stability of the covariance matrix and affect model accuracy [89]. This may also account for the modest effect sizes observed in the serial mediation models (e.g., β = 0.03), limiting their practical significance. Future research should employ larger, more targeted samples to enhance the validity and generalizability of the findings.

## 6. Conclusions

Grounded in status quo bias theory, this study investigated the intricate relationship between misinformation recognition and fact-checking intent among Chinese young adults, a topic that remains largely underexplored in the context of oral health misinformation, particularly within collectivist societies. To address this gap, a stimulus-based, systematic survey method was employed. A valid sample (N = 452) was recruited through an online questionnaire focused on oral health-related content. The study sequentially examined the reliability and validity of each measurement and tested direct, mediated, and serially mediated hypotheses using SPSS 26.0 and Jamovi 2.6.24. The findings offer valuable insights into the underlying mechanisms associated with the relationship between misinformation recognition and fact-checking intent within the oral health domain among this demographic. Potential implications are discussed for public health departments and professionals, with the goal of enhancing awareness of oral health misinformation and promoting greater engagement in fact-checking behaviors in the field of oral health.

## Figures and Tables

**Table 1:** Overview of misinformation video stimuli.

Stimulus	Main Content	Misinformation Theme	Duration
1	Shares a method for making a homemade solution for cleaning teeth, allowing you to avoid the cost of professional cleaning.	DIY remedy	20 s
2	Promotes an opportunity to get full dental implant service at a very low price.	Cost-related deception	37 s

**Table 2:** Fornell–Larcker criterion for discriminant validity.

Variables	1	2	3	4	5
MR	0.89				
NFC	0.13 **	0.87			
PS	0.24 **	0.29 **	0.84		
RSQB	0.13 **	0.54 **	0.40 **	0.85	
FCI	0.02	0.53 **	0.35 **	0.70 **	0.72

** *p* < 0.01, MR = misinformation recognition, NFC = need for cognition, PS = perceived seriousness, RSQB = reversed status quo bias, FCI = fact-checking intent.

**Table 3:** HTMT for discriminant validity.

Variables	1	2	3	4	5
MR					
NFC	0.15				
PS	0.27	0.31			
RSQB	0.16	0.67	0.48		
FCI	0.02	0.64	0.36	0.72	

MR = misinformation recognition, NFC = need for cognition, PS = perceived seriousness, RSQB = reversed status quo bias, FCI = fact-checking intent.

**Table 4:** Demographic characteristics of the participants (N = 452).

Variable	Item	Count	Percentage
Sex	Female	243	53.8%
	Male	209	46.2%
Education level	High school	23	5.1%
	Undergraduate	318	70.4%
	Postgraduates	111	24.6%
Age	18–23 years old	292	64.6%
	24–28 years old	106	23.5%
	29–36 years old	54	11.9%
Monthly household income (CNY)	1000–3999	119	26.3%
	4000–8999	99	21.9%
	9000–13,999	131	29.0%
	≥14,000	103	22.8%
	Total	452	100%

**Table 5:** Summary of the mediated effects among variables.

Mediated Effects	Effect	P	LLCI	ULCI
MR → NFC →FCI	0.10	***	0.01	0.10
MR → PS →FCI	0.10	***	0.04	0.11
MR → NFC → RSQB → FCI	0.03	***	0.01	0.10
MR → PS → RSQB → FCI	0.10	***	0.02	0.10

*** *p* < 0.001, MR = misinformation recognition, NFC = need for cognition, PS = perceived seriousness, RSQB = reversed status quo bias, FCI = fact-checking intent.

**Table 6:** Overview of hypothesis testing results.

Hypotheses	Relationship	Result
Hypothesis 1	MR is positively associated with Chinese college young adults’ NFC.	Supported
Hypothesis 2	MR is positively associated with Chinese college young adults’ PS.	Supported
Hypothesis 3	NFC mediates the relationship between MR and Chinese young adults’ FCI.	Supported
Hypothesis 4	PS mediates the relationship between MR and Chinese young adults’ FCI.	Supported
Hypothesis 5	NFC and RSQB play a serial mediating role in the relationship between MR and Chinese young adults’ FCI.	Supported
Hypothesis 6	PS and RSQB play a serial mediating role in the relationship between MR and Chinese young adults’ FCI.	Supported

MR = misinformation recognition, NFC = need for cognition, PS = perceived seriousness, RSQB = reversed status quo bias, FCI = fact-checking intent.

## Data Availability

The data supporting the findings of this study are available upon reasonable request from the corresponding author, subject to compliance with institutional data-sharing policies and applicable privacy or ethical restrictions.

## Supplementary Materials

The following supporting information can be downloaded at: https://www.mdpi.com/article/10.3390/healthcare13111354/s1, Table S1: Checklist for Reporting Results of Internet E-Surveys (CHERRIES).

## Appendix A

**Table A1:** Factor loadings, composite reliability (CR), and average variance extracted (AVE) for each item.

Construct	Source	Items	Loading	α	CR	AVE
MR	[59,60]	I think the contents are inaccurate.	0.89	0.92	0.98	0.81
		I think the sources of these contents are unreliable.	0.91			
		I think the providers of these contents have low credibility.	0.91			
		Regardless of accuracy, I find these videos suspicious.	0.88			
NFC	[43]	I am willing to give oral health information a lot of thought, even if it takes a long time.	0.80	0.83	0.95	0.76
		Compared to others, I spend more time thinking about oral health information.	0.90			
		I try to make myself think more about issues related to oral health.	0.91			
PS	[61]	Believing in misinformation would be bad for my health.	0.83	0.86	00.97	0.71
		Believing in misinformation would cause me financial loss.	0.84			
		Believing in misinformation would reduce my work efficiency.	0.81			
		Believing in misinformation would lower my quality of life.	0.88			
RSQB	[62,63]	I generally consider suspicious information carefully and actively fact-check it online.	0.84	0.87	0.97	0.73
		I am very willing to proactively fact-check suspicious information.	0.88			
		I think it is important to fact-check suspicious information.	0.86			
		When people encourage me to fact-check suspicious information, I willingly do so.	0.83			
FCI	[64]	I would fact-check information on short video platforms.	0.70	0.80	0.97	0.52
		I would fact-check information through major news organizations.	0.80			
		I would fact-check information by consulting friends and family.	0.70			
		I would fact-check information using search engines (e.g., Baidu, Google).	0.70			
		I would fact-check information through social media (e.g., Weibo, WeChat).	0.71			
		I would fact-check information by referring to other sources (e.g., expert opinions, government websites).	0.70			
