# Supplementary material for: Unpacking Young Adults’ Fact-Checking Intent on Oral Health Misinformation: Parallel Mediating Roles of Need for Cognition and Perceived Seriousness—A Cross-Sectional Study
Source: Healthcare (Basel). 2025 Jun 5;13(11):1354. doi: 10.3390/healthcare13111354 (PMC12155137; doi:10.3390/healthcare13111354)
Supplement: Supplementary file 1 [file healthcare-13-01354-s001.zip › healthcare-3576021-supplementary.pdf]

Table S1. Checklist for Reporting Results of Internet E-Surveys (CHERRIES)

Categories	Checklist Item	Description
Design	Describe survey design	The target population comprised young adults aged 18 to 36 who actively use major social media platforms such as WeChat, Douyin, and Xiaohongshu.
IRB approval	IRB approval	Ethical approval for the study was granted by the Academic Committee of the School of Journalism and Communication at Central China Normal University (CC20250120, 20 January 2025)
	Informed consent	Informed consent was obtained at the start of the online questionnaire, with assurances of confidentiality and the right to withdraw provided to all participants.
	Data protection	In compliance with relevant data protection regulations and the policies of Tencent Questionnaires, all data were securely managed and stored. No personally identifiable information, such as phone numbers or email addresses, was collected, ensuring participant anonymity and confidentiality throughout the study.
Development and testing	Development	Survey measures were adapted from established instruments in prior studies. Key modifications included independent translation into Chinese by three language experts and evaluation by two public health experts to ensure linguistic accuracy and conceptual validity.
	Testing	Sixty-five volunteers (aged 18 to 36) were recruited to conduct a pilot study. Based on their feedback, ambiguous survey items were removed. The results indicated acceptable internal consistency, and the structural integrity met the required standards.
Participant recruitment and access to the questionnaire	Open survey versus closed survey	This was an open survey.
	Contact mode	Participants were recruited through Tencent Questionnaires, an online survey platform. Access to the questionnaire was granted only to individuals who confirmed they had read the written informed consent on the first page.
	Advertising the survey	Professionals from Tencent Questionnaires facilitated the recruitment process as part of their official responsibilities.
	Web/E-mail	The online questionnaire was distributed via Tencent Questionnaires (https://wj.qq.com/, accessed on January 25, 2025), and responses were automatically collected through the platform.

Survey administration	Context	The questionnaire links were distributed by Tencent Questionnaires to a verified pool of over 48 million Chinese individuals. The links included only information relevant to the current study.
	Mandatory/ voluntary	Voluntary.
	Incentives	Participants received compensation for their participation, as facilitated through Tencent Questionnaires' integrated incentive distribution system.
	Time/Date	Recruitment began on January 25, 2025, and ended on March 10, 2025.
	Randomization of items or questionnaires	No randomisation of items was used.
	Adaptive questioning	Not employed in this survey.
	Number of Items	30
	Number of screens (pages)	4
	Completeness check	All questions in the questionnaire were mandatory; therefore, respondents could not submit the survey without completing every item.
	Review step	The survey interface included navigation buttons, enabling respondents to review and revise their responses prior to submission.
Response rates	Unique site visitor	This feature was not implemented in the present survey.
	View rate (Ratio of unique survey visitors/unique site visitors)	Not applicable
	Participation rate (Ratio of unique visitors who agreed to participate/unique first survey page visitors)	A total of 510 Chinese young adults were invited to participate, and 482 individuals completed the survey.
	Completion rate (Ratio of users who finished the survey/users who agreed to participate)	Following data cleaning, 30 responses were excluded due to completion times under 120 seconds and evidence of straight-line response patterns. The final analytical sample comprised 452 valid responses.
Preventing multiple entries from the same individual	Cookies used	Not employed in this survey.
	IP check	Not employed in this survey.
	Log file analysis	Not employed in this survey.
	Registration	Not employed in this survey.

Analysis	Handling of incomplete questionnaires	Incomplete questionnaires were automatically excluded from the dataset.
	Questionnaires submitted with an atypical timestamp	Not employed in this survey.
	Statistical correction	Not applicable
